# Breakfast can Affect Routine Hematology and Coagulation Laboratory Testing: An Evaluation on Behalf of COLABIOCLI WG-PRE-LATAM

**DOI:** 10.1055/s-0039-3401002

**Published:** 2019-12-17

**Authors:** Maria Elena Arredondo, Eduardo Aranda, Rubén Astorga, Lorena Michele Brennan-Bourdon, Marise Danielle Campelo, Silvia Flores, Claudio Medel, Ignacio Manríquez, Patricia Ochoa, Beatriz Varela, Carlos Vega Salinas, Gabriel Lima-Oliveira

**Affiliations:** 1 BIONET S.A., Santiago, Chile; 2 Laboratory of Thrombosis and Hemostasis, Department of Hematology-Oncology, School of Medicine, Pontificia Universidad Católica de Chile, Santiago, Chile; 3 Clinical Laboratory Network from the State of Jalisco, Public Health State Laboratory (LESP), Comisión Para la Protección Contra Riesgos Sanitarios del Estado de Jalisco (COPRISJAL), Guadalajara, Mexico; 4 Clinical Laboratory Bioanalise, Teresina, Piaui, Brazil; 5 Universidad Peruana Cayetano Heredia, Lima, Perú; 6 Clínica Dávila y Servicios Médicos S.A., Santiago, Chile; 7 Facultad de Medicina, Universidad Católica de Cuenca, Cuenca, Ecuador; 8 LAC, Montevideo, Uruguay; 9 Section of Clinical Biochemistry, Department of Neurosciences, Biomedicine and Movement Sciences, University of Verona, Verona, Italy

**Keywords:** reproducibility of results, postprandial period, fasting, diagnostic errors, blood specimen collection

## Abstract

Laboratories worldwide perform both hematological and coagulation testing on patients avoiding fasting time. In 2017, the Latin America Confederation of Clinical Biochemistry (COLABIOCLI) commissioned the Latin American Working Group for Preanalytical Phase (WG-PRE-LATAM) to study preanalytical variability and establish guidelines for preanalytical procedures to be applied by clinical laboratories and health care professionals. This study, on behalf of COLABIOCLI WG-PRE-LATAM, aims to evaluate the effect of the breakfast on routine hematology and coagulation laboratory testing. We studied 20 healthy volunteers who consumed a breakfast containing a standardized amount of carbohydrates, proteins, and lipids. We collected blood specimens for routine hematology and coagulation laboratory testing before breakfast and 1, 2, and 4 hours thereafter. Significant differences between samples were assessed by the Wilcoxon ranked-pairs test. Statistically significant differences ( *p* < 0.05) between basal and 4 hours after the breakfast were observed for red blood cells, hemoglobin, hematocrit, mean corpuscular volume, white blood cells, neutrophils, lymphocytes, monocytes, mean platelet volume, and activated partial thromboplastin time. In conclusion, the significant variations observed in several hematological parameters, and activated partial thromboplastin time due to breakfast feeding demonstrate that the fasting time needs to be carefully considered prior to performing routine hematological and coagulation testing to avoid interpretive mistakes of test results, and to guarantee patient safety. Therefore, COLABIOCLI WG-PRE-LATAM encourages laboratory quality managers to standardize the fasting requirements in their laboratory, i.e., 12 hours.

## Introduction

In 2017, the Latin America Confederation of Clinical Biochemistry (COLABIOCLI) commissioned the Latin American Working Group for Preanalytical Phase (WG-PRE-LATAM) to study preanalytical variability and establish guidelines for preanalytical procedures to be applied by clinical laboratories and health care professionals. The procedures and processes involved in the preanalytical phase are considered the main source of laboratory variability. 1 2 Providing proper information for patient preparation before laboratory testing is one important issue addressed to accredited laboratories to guarantee patient safety. 3

Lippi et al, 9 years ago (2010), evaluated the impact of an Italian light meal on hematology testing and evidenced significant variation of several hematological parameters. Therefore, to interpret the results of hematological tests correctly, the fasting time needs to be carefully considered. 4 However, laboratories worldwide perform both hematological and coagulation testing on patients avoiding fasting time.

This study, on behalf of COLABIOCLI WG-PRE-LATAM, aims to evaluate the effect of the breakfast on routine hematology and coagulation laboratory testing.

## Materials and Methods

A total of 20 healthy volunteers (10 women and 10 men; average age was 42 [23–64] years) were selected from the personnel of the Laboratory BioNet (Santiago, Chile) and included in the study. Informed consent was obtained from all study subjects according to the 2013 Declaration of Helsinki and the protocol was approved by the ethics committee.

A single expert phlebotomist, following the international EFLM-COLABIOCLI recommendations, 5 performed all venous blood sampling procedures. To eliminate possible blood distribution interferences, all volunteers stayed sitting for 15 minutes. 6 7 A subcutaneous tissue transilluminator device (Venoscópio IV plus; Duan do Brasil, Brazil) was used to locate a vein on the forearm to prevent venous stasis interference from the use of the tourniquet, 8 9 10 and avoid clench. 11 12

All blood samples were collected respectively into one 3.6-mL evacuated tube containing sodium citrate 3.2%, one 3.0-mL evacuated tube containing K _2_ EDTA, and one 3.0-mL evacuated tube containing K _3_ EDTA (Vacumed, FL Medical, Torreglia, Italy) using a 20-gauge needle (FL Medical). To eliminate any possible interference due to either the contact phase or tissue factor, approximately 2 mL of blood were preliminarily collected in a discard tube without additive. The first blood sample was collected between 8:00 a.m. and 8:30 a.m. after a 12-hour overnight fast (i.e., volunteers were not allowed to eat, or drink during this period of time). Moreover, strenuous physical activity was avoided by all volunteers at least 72 hours before blood collection. Volunteers were not smokers and had not intake any medication. Immediately after the first venous blood sampling procedure, the volunteers consumed the breakfast, containing standardized amounts of carbohydrates, protein, and lipids. Table 1 shows the exact composition of the breakfast. Subsequent venous blood samplings were performed at 1, 2, and 4 hours after breakfast. Each phase of the blood sampling procedure was appropriately standardized, including the use of needles and evacuated tubes from the same type and lot number. No specimens were discarded due to unsatisfactory attempts, for example, problems in locating a suitable vein.

**Table 1:** Nutritional composition of Chilean breakfast

Nutritional composition	Sandwich	Chocolate snack	Yogurt	Orange juice	Total
Number (overall weight)	1 (130 g)	1 (45 g)	1 (120 g)	1 (200 mL)	495 g
Kcal	384	241	103	50	778
Kilojoule (KJ)	1,607	1,008	431	209	3,255
Protein (g)	17.6	2.8	3.2	0.6	24.2
Carbohydrate (g)	50.3	27.0	19.0	11.2	107.5
Total lipids (g)	12.5	14.0	1.6	0.2	28.3
Cholesterol (mg)	19.2	NA	NA	NA	19.2

Abbreviation: NA, not available.

The sample tubes for coagulation assays were left in upright position for 30 minutes at room temperature (20°C) to ensure complete blood stability before centrifugation. Then, sample tubes were centrifuged at 1,500 × *g* for 15 minutes at room temperature, according to the instructions provided by the evacuated tube manufacturer (i.e., FL Medical). No samples were either hemolytic or lipemic by visual inspection.

All samples collected were assayed in a single analytical run with the same analyzer according to the manufacturer's specifications and using proprietary reagents. The panel of tests performed is shown in Tables 2 and 3 . Hematological parameters were assayed on ADIVIA 2120 (Siemens Healthcare Diagnostics Inc., Tarrytown, New York, United States), and on Sysmex XN-1000 (Sysmex Corporation, Kobe, Japan), whereas coagulation tests were performed on ACL TOP 700 (Werfen, Barcelona, Spain) and on Sysmex CA-1500 (Sysmex Corporation). The instruments were calibrated against appropriate proprietary reference standard materials and verified with independent third-party control materials from calibrator materials, as recommended. 13 The evaluation of the within-run precision by the internal quality control of the instruments used, showed low coefficients of variation ( Tables 2 and 3 ).

**Table 2:** Postprandial variation on complete blood count after Chilean breakfast

Parameters	K _2_ EDTA				K _3_ EDTA			
	Basal	1 h	2 h	4 h	Basal	1 h	2 h	4 h
RBC (10 ^12^ /L) ^a^ (CVa = 1.05%)	4.69 [4.50–4.98]	4.69 [4.53–5.05]	4.67 [4.51–4.99]	4.66 [4.52–4.93]	4.76 [4.54–5.01]	4.73 [4.58–5.07]	4.62 [4.47–5.01]	4.60 [4.49–4.93]
*p* -Value		0.153	0.081	**0.014**		0.940	< **0.001**	< **0.001**
*p* -Value K _2_ vs. K _3_	**0.007**							
RBC (10 ^12^ /L) ^b^ (CVa = 0.58%)	4.76 [4.55–5.47]	4.75 [4.61–5.46]	4.70 [4.56–5.39]	4.66 [4.50–5.32]	4.76 [4.53–5.45]	4.76 [4.59–5.44]	4.72 [4.53–5.33]	4.68 [4.50–5.39]
*p* -Value		0.780	**0.012**	< **0.001**		1.000	**0.012**	**0.002**
*p* -Value K _2_ vs. K _3_	0.709							
Retic (10 ^9^ /L) ^a^ (CVa = 4.07%)	85.8 [75.1–106]	91.4 [77.6–108]	87.5 [71.4–101]	95.0 [71.0–97.4]	83.9 [71.8–105]	85.4 [73.8–104]	86.2 [69.3–94.7]	87.0 [66.8–100]
*p* -Value		0.185	0.668	0.926		0.940	**0.003**	0.097
*p* -Value K _2_ vs. K _3_	0.985							
Retic (10 ^9^ /L) ^b^ (CVa = 3.21%)	53.5 [46.8–73.0]	56.6 [46.2–69.6]	51.2 [46.4–68.8]	53.8 [46.4–72.8]	56.4 [45.1–76.3]	54.0 [47.4–72.5]	52.1 [45.8–73.0]	53.4 [45.2–69.3]
*p* -Value		0.103	0.438	0.426		0.330	0.209	0.116
*p* -Value K _2_ vs. K _3_	0.670							
Hb (g/L) ^a^ (CVa = 0.93%)	146 [138–157]	144 [139–154]	144 [136–154]	142 [134–152]	144 [138–153]	145 [139–156]	142 [134–153]	142 [135–152]
*p* -Value		0.549	**0.004**	**< 0.001**		0.699	**0.002**	**< 0.001**
*p* -Value K _2_ vs. K _3_	**0.043**							
Hb (g/L) ^b^ (CVa = 0.53%)	147 [140–160]	145 [142–160]	145 [139–156]	143 [140–156]	146 [142–161]	146 [142–159]	144 [138–156]	143 [139–157]
*p* -Value		0.406	**0.042**	**0.006**		0.875	**0.008**	**0.002**
*p* -Value K _2_ vs. K _3_	0.944							
Hct (%) ^a^ (CVa = 1.08%)	43.6 [41.8–46.4]	43.7 [42.2–46.8]	43.6 [41.3–46.9]	42.9 [41.0–45.4]	43.4 [41.8–45.9]	43.6 [41.6–46.6]	42.2 [40.4–45.6]	42.1 [40.6–44.9]
*p* -Value		**0.038**	**0.018**	**< 0.001**		0.765	**0.001**	**< 0.001**
*p* -Value K _2_ vs. K _3_	**0.013**							
Hct (%) ^b^ (CVa = 0.54%)	45.8 [43.8–49.7]	45.3 [44.2–49.4]	44.6 [42.5–48.6]	43.8 [41.9–47.6]	44.4 [42.7–48.6]	44.4 [43.2–48.1]	43.4 [41.4–47.2]	42.7 [41.5–47.0]
*p* -Value		0.683	**0.003**	**0.001**		1.000	**0.002**	**0.001**
*p* -Value K _2_ vs. K _3_	**0.001**							
MCV (fL) ^a^ (CVa = 0.32%)	92.8 [90.0–95.6]	92.8 [90.2–95.1]	92.2 [89.8–94.4]	91.6 [89.5–94.1]	91.5 [88.9–94.1]	91.4 [89.0–93.6]	90.9 [88.7–92.9]	90.3 [88.0–92.5]
*p* -Value		0.059	**< 0.001**	**< 0.001**		0.093	**< 0.001**	**< 0.001**
*p* -Value K _2_ vs. K _3_	**< 0.001**							
MCV (fL) ^b^ (CVa = 0.14%)	95.0 [91.1–96.9]	95.2 [91.1–96.4]	93.8 [90.3–96.0]	93.0 [90.0–95.1]	93.2 [89.4–94.6]	93.2 [89.3–94.3]	91.8 [88.4–93.8]	91.4 [88.0–93.2]
*p* -Value		0.306	**0.004**	**< 0.001**		0.937	0.002	0.001
*p* -Value K _2_ vs. K _3_	**0.001**							
RDW (%) ^a^ (CVa = 0.57%)	13.0 [12.6–13.3]	13.0 [12.5–13.3]	12.9 [12.5–13.3]	13.0 [12.4–13.2]	13.0 [12.5–13.3]	13.0 [12.4–13.3]	12.9 [12.5–13.2]	12.9 [12.4–13.2]
*p* -Value		0.479	0.064	0.592		0.461	0.079	0.077
*p* -Value K _2_ vs. K _3_	0.745							
RDW (%) ^b^ (CVa = 0.56%)	13.2 [12.7–13.7]	13.2 [12.8–13.6]	13.0 [12.7–13.5]	13.0 [12.7–13.5]	13.1 [12.7–13.6]	13.0 [12.8–13.5]	13.0 [12.7–13.4]	13.0 [12.5–13.4]
*p* -Value		0.507	**0.012**	**0.011**		0.546	0.586	0.588
*p* -Value K _2_ vs. K _3_	0.114							
WBC (10 ^9^ /L) ^a^ (CVa = 1.66%)	6.09 [5.15–7.06]	6.20 [5.25–7.92]	6.80 [5.87–8.05]	7.12 [6.11–8.82]	6.36 [5.14–7.10]	6.44 [5.55–7.99]	6.72 [5.86–8.16]	7.18 [6.15–8.94]
*p* -Value		0.422	**0.002**	**< 0.001**		0.287	**0.004**	**0.001**
*p* -Value K _2_ vs. K _3_	0.179							
WBC (10 ^9^ /L) ^b^ (CVa = 1.30%)	5.99 [4.93–6.90]	6.27 [5.54–6.71]	6.53 [6.07–7.65]	6.97 [6.50–8.51]	6.03 [4.92–6.86]	6.32 [5.56–6.64]	6.73 [6.12–7.91]	7.05 [6.46–8.43]
*p* -Value		0.119	**0.003**	**< 0.001**		0.194	**0.001**	**0.001**
*p* -Value K _2_ vs. K _3_	0.834							
NEU (10 ^9^ /L) ^a^ (CVa = 2.27%)	3.45 [2.46–4.10]	3.66 [3.01–4.77]	4.12 [3.19–4.88]	4.06 [3.10–5.03]	3.52 [2.41–4.19]	3.76 [3.05–5.03]	4.18 [3.18–4.92]	4.12 [3.23–5.08]
*p* -Value		**0.001**	**0.001**	**0.002**		**0.001**	**0.001**	**0.002**
*p* -Value K _2_ vs. K _3_	**0.023**							
NEU (10 ^9^ /L) ^b^ (CVa = 2.44%)	3.27 [2.32–3.83]	3.60 [2.82–4.08]	4.00 [3.03–4.66]	4.04 [2.94–5.16]	3.28 [2.39–3.86]	3.72 [2.90–4.17]	3.98 [2.98–4.82]	4.18 [2.88–5.15]
*p* -Value		**0.003**	**0.001**	**0.002**		**0.003**	**0.002**	**< 0.001**
*p* -Value K _2_ vs. K _3_	0.571							
LYMP (10 ^9^ /L) ^a^ (CVa = 3.74%)	2.04 [1.76–2.42]	1.87 [1.45–2.20]	1.91 [1.68–2.36]	2.30 [2.03–2.78]	2.01 [1.80–2.39]	1.86 [1.43–2.22]	1.90 [1.66–2.38]	2.30 [1.97–2.83]
*p* -Value		**0.013**	0.629	**< 0.001**		**0.016**	0.906	**< 0.001**
*p* -Value K _2_ vs. K _3_	0.663							
LYMP (10 ^9^ /L) ^b^ (CVa = 1.44%)	2.07 [1.76–2.32]	1.98 [1.49–2.28]	2.06 [1.83–2.50]	2.40 [2.18–2.78]	2.04 [1.80–2.39]	1.94 [1.44–2.24]	2.13 [1.86–2.52]	2.22 [2.15–2.88]
*p* -Value		0.132	0.543	**< 0.001**		0.119	0.315	**< 0.001**
*p* -Value K _2_ vs. K _3_	0.551							
Mono (10 ^9^ /L) ^a^ (CVa = 6.27%)	0.34 [0.29–0.43]	0.32 [0.26–0.36]	0.36 [0.32–0.42]	0.36 [0.33–0.42]	0.36 [0.32–0.44]	0.32 [0.29–0.42]	0.36 [0.32–0.42]	0.38 [0.33–0.42]
*p* -Value		**0.014**	**0.022**	**0.024**		0.139	0.920	0.170
*p* -Value K _2_ vs. K _3_	0.217							
Mono (10 ^9^ /L) ^b^ (CVa = 4.34%)	0.44 [0.40–0.53]	0.40 [0.34–0.47]	0.46 [0.41–0.56]	0.52 [0.42–0.57]	0.40 [0.36–0.53]	0.36 [0.32–0.47]	0.49 [0.39–0.57]	0.50 [0.39–0.59]
*p* -Value		0.052	0.221	**0.004**		**0.220**	**0.033**	**0.007**
*p* -Value K _2_ vs. K _3_	**0.021**							
Eos (10 ^9^ /L) ^a^ (CVa = 9.10%)	0.20 [0.11–0.27]	0.18 [0.10–0.24]	0.20 [0.10–0.28]	0.22 [0.10–0.25]	0.22 [0.11–0.28]	0.20 [0.10–0.28]	0.18 [0.10–0.26]	0.20 [0.10–0.26]
*p* -Value		0.161	0.499	1.000		0.147	0.053	0.528
*p* -Value K _2_ vs. K _3_	0.300							
Eos (10 ^9^ /L) ^b^ (CVa = 8.18%)	0.22 [0.11–0.32]	0.20 [0.07–0.26]	0.23 [0.07–0.28]	0.21 [0.08–0.26]	0.20 [0.10–0.30]	0.20 [0.06–2.25]	0.23 [0.06–0.29]	0.22 [0.07–0.30]
*p* -Value		0.248	0.550	0.683		0.184	0.476	0.753
*p* -Value K _2_ vs. K _3_	0.784							
Baso (10 ^9^ /L) ^a^ (CVa = 15.2%)	0.04 [0.03–0.05]	0.04 [0.03–0.05]	0.04 [0.03–0.05]	0.04 [0.03–0.05]	0.04 [0.03–0.05]	0.04 [0.04–0.05]	0.04 [0.03–0.05]	0.04 [0.03–0.05]
*p* -Value		0.264	0.229	0.219		0.796	0.954	0.837
*p* -Value K _2_ vs. K _3_	0.209							
Baso (10 ^9^ /L) ^b^ (CVa = 18.3%)	0.04 [0.03–0.05]	0.04 [0.03–0.04]	0.04 [0.03–0.05]	0.04 [0.03–0.06]	0.04 [0.03–0.05]	0.04 [0.03–0.06]	0.04 [0.04–0.05]	0.04 [0.03–0.05]
*p* -Value		0.722	0.611	0.548		0.781	0.257	0.187
*p* -Value K _2_ vs. K _3_	1.000							
Luc (10 ^9^ /L) ^a^ (CVa = 11.5%)	0.11 [0.09–0.12]	0.09 [0.07–0.10]	0.10 [0.08–0.12]	0.13 [0.11–0.15]	0.09 [0.09–0.10]	0.08 [0.06–0.09]	0.09 [0.08–0.11]	0.12 [0.10–0.13]
*p* -Value		**0.002**	0.244	**0.002**		**0.017**	0.417	**0.004**
*p* -Value K _2_ vs. K _3_	**0.003**							
IG (10 ^9^ /L) ^b^ (CVa = 35.1%)	0.02 [0.01–0.03]	0.02 [0.02–0.03]	0.03 [0.02–0.04]	0.02 [0.02–0.04]	0.02 [0.01–0.03]	0.02 [0.02–0.03]	0.02 [0.02–0.03]	0.02 [0.02–0.03]
*p* -Value		0.608	0.053	0.061		0.111	0.674	0.193
*p* -Value K _2_ vs. K _3_	0.803							
PLT (10 ^9^ /L) ^a^ (CVa = 2.43%)	274 [240–323]	278 [247–332]	273 [246–323]	266 [253–328]	261 [245–331]	273 [247–324]	275 [245–327]	270 [253–322]
*p* -Value		**0.002**	0.057	**0.016**		0.100	0.332	0.093
*p* -Value K _2_ vs. K _3_	0.370							
PLT (10 ^9^ /L) ^b^ (CVa = 0.66%)	247 [205–301]	264 [217–305]	254 [213–303]	252 [222–304]	256 [210–300]	258 [222–311]	258 [216–307]	258 [224–311]
*p* -Value		**0.006**	**0.027**	0.109		0.258	0.826	0.456
*p* -Value K _2_ vs. K _3_	0.051							
MPV (fL) ^a^ (CVa = 1.88%)	8.20 [7.52–9.00]	7.95 [7.42–8.68]	7.65 [7.20–8.50]	7.65 [7.02–8.65]	8.25 [7.40–8.78]	7.85 [7.35–8.75]	7.60 [7.22–8.58]	7.55 [7.02–8.48]
*p* -Value		**< 0.001**	**< 0.001**	**< 0.001**		**0.024**	**< 0.001**	**< 0.001**
*p* -Value K _2_ vs. K _3_	0.458							
MPV (fL) ^b^ (CVa = 0.87%)	9.85 [9.08–10.8]	9.75 [8.98–10.8]	9.80 [9.08–10.8]	9.90 [9.08–10.9]	10.2 [9.28–11.0]	10.1 [9.18–11.0]	10.1 [9.40–11.0]	10.2 [9.28–11.0]
*p* -Value		0.784	1.000	0.064		0.436	1.000	1.000
*p* -Value K _2_ vs. K _3_	**0.001**							

Abbreviations: Baso, basophils; CVa, analytical coefficient of variation; Eos, eosinophils; Hb, hemoglobin; Hct, hematocrit; IG, immature granulocyte; Luc, large unstained cells; LYMP, lymphocytes; MCV, mean corpuscular volume; Mono, monocytes; MPV, mean platelet volume; NEU, neutrophils; PLT, platelet count; RBC, red blood cell count; RDW, RBC distribution width; Retic, reticulocyte; WBC, white blood cell count.

a Hematological parameter was assayed on ADIVIA 2120 (Siemens Healthcare Diagnostics Inc, Tarrytown, New York, United States).

b Hematological parameter was assayed on Sysmex XN-1000 (Sysmex Corporation, Kobe, Japan).

Note: Results are presented as median [interquartile range]. *p* -Value represents the significance by Wilcoxon ranked-pairs test. Bold *p* -values indicate statistical significance ( *p* < 0.05).

**Table 3:** Postprandial variation on routine coagulation testing after Chilean breakfast

Tests	Basal	1 h	2 h	4 h
aPTT (s) ^a^ (CVa = 1.56%)	28.9 [27.8–29.9]	29.2 [27.8–30.5]	27.6 [27.8–30.2]	29.2 [28.0–30.2]
*p* -Value		0.111	**0.034**	**0.037**
aPTT (s) ^b^ (CVa = 5.04%)	34.4 [33.0–36.4]	34.6 [32.7–36.2]	33.4 [32.6–35.1]	33.9 [32.3–34.9]
*p* -Value		0.334	**0.033**	**0.007**
PT (s) ^a^ (CVa = 1.73%)	11.2 [10.6–11.5]	11.2 [10.7–11.4]	11.0 [10.6–11.4]	11.0 [10.6–11.3]
*p* -Value		0.693	0.704	0.307
PT (s) ^b^ (CVa = 2.00%)	11.2 [11.1–11.5]	11.3 [11.0–11.4]	11.0 [11.0–11.4]	11.0 [10.9–11.2]
*p* -Value		0.220	0.069	0.097
Fib (mg/dL) ^a^ (CVa = 4.50%)	304 [285–354]	304 [294–351]	302 [281–340]	306 [289–350]
*p* -Value		0.571	**0.010**	0.687
Fib (mg/dL) ^b^ (CVa = 7.19%)	254 [226–288]	254 [231–286]	246 [223–279]	254 [225–289]
*p* -Value		0.552	**< 0.001**	0.332
AT (%) ^a^ (CVa = 2.60%)	112 [106–116]	112 [108–118]	112 [106–118]	114 [106–121]
*p* -Value		0.389	0.050	**0.004**
PC (%) ^a^ (CVa = 1.40%)	102 [93.0–124]	103 [96–128]	103 [94.5–124]	102 [95.8–132]
*p* -Value		0.143	0.217	0.819
Free PS (%) ^a^ (CVa = 6.51)	103 [93.7–108]	103 [95.4–108]	106 [95.2–107]	106 [99.6–110]
*p* -Value		0.542	**< 0.001**	**< 0.001**

Abbreviations: aPTT, activated partial thromboplastin time; AT, antithrombin; CVa, analytical coefficient of variation; Fib, fibrinogen; PC, protein C; PS, protein S; PT, prothrombin time.

a Coagulation test was performed on ACL TOP 700 (Werfen, Barcelona, Spain).

b Coagulation test was performed on Sysmex CA-1500 (Sysmex Corporation).

Note: Results are presented as median [interquartile range]. *p* -Value represents the significance by Wilcoxon ranked-pairs test. Bold *p* -values indicate statistical significance ( *p* < 0.05).

### Statistical Analysis

To assess statistical differences between samples, the Wilcoxon ranked-pairs test was used in agreement with Simundic's 14 recommendations regarding sample size (i.e., less than 30 samples), with a licensed statistical software (GraphPad Prism version 5.01, La Jolla, California, United States). The level of statistical significance was set at *p* < 0.05. The mean percentage difference in each test parameter with statistical significance was calculated using the formula:

Mean percentage difference = [(× h after breakfast − basal)/× h after breakfast] × 100

Finally, the mean percentage differences from blood samples at 1, 2, and 4 hours after breakfast were compared with the desirable specifications for imprecision (DSI) derived from biological variation. 15 DSI was used as our criterion of acceptance in lipemia analytical interference testing, then interferograms were provided for each laboratory parameter with a significant difference between basal and × h after the breakfast.

## Results

**Fig. 1:**
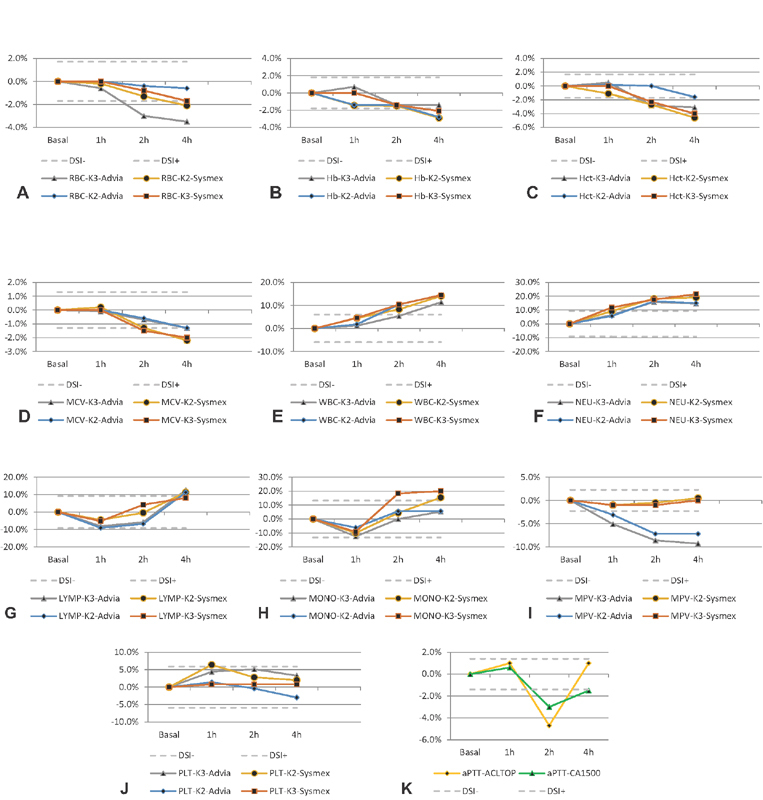
Interferograms. ( **A** ) red blood cell (RBC); ( **B** ) hemoglobin (Hb); ( **C** ) hematocrit (Hct); ( **D** ) mean corpuscular volume (MCV); ( **E** ) white blood cell (WBC); ( **F** ) neutrophil (NEU); ( **G** ) lymphocyte (LYMP); ( **H** ) monocyte (MONO); ( **I** ) mean platelet volume (MPV); ( **J** ) platelet (PLT); and ( **K** ) activated partial thromboplastin time (aPTT). Hours after the breakfast ( *x* -axis) are plotted against bias values ( *y* -axis). Solid line, bias. Dashed lines, acceptable criteria based on desirable specification for imprecision (DSI) derived from biologic variation.

The results of this investigation are presented as median (interquartile range) in Tables 2 and 3 . Statistically significant differences ( *p* < 0.05) between basal and 4 hours after the breakfast were observed for red blood cell (RBC), hemoglobin (Hb), hematocrit (Hct), mean corpuscular volume (MCV), white blood cell (WBC), neutrophil (NEU), lymphocyte (LYMP), monocyte (MONO), mean platelet volume (MPV), and activated partial thromboplastin time (aPTT) ( Fig. 1 ). In regard to K _2_ EDTA versus K _3_ EDTA, statistically significant differences ( *p* < 0.05) were observed for RBC, Hb, NEU, and Luc in samples assayed on Advia 2120 (Siemens Healthcare, GmbH); MONO and MPV in samples tested on Sysmex XN-1000 (Sysmex Corporation), whereas Hct and MCV values were different in both instruments ( Table 2 ; Fig. 1 ).

## Discussion

Significant decreases were observed for RBC, Hb, Hct, and MCV 2 hours after breakfast and no return to baseline in the following 4 hours after food intake ( Table 2 , Fig. 1A–D ). A study performed in starved rats reported increases in Hb, Hct, and RBC counts because of the hemoconcentration/dehydration effects caused by the greatly reduced water intake during fasting time. 16 Consequently, we can explain our RBC, Hb, and Hct results by the hemodiluition/rehydration of the volunteers during breakfast, since they consumed 200 mL of orange juice and 120 g of yogurt ( Table 1 ). An unanswered question is whether subjects who consume fixed volume of plain water 2 or 4 hours before blood collection impact on this kind of variability. A report by Unger et al, evaluated the effect of water ingestion (300 mL) 1 hour before blood collection, concluding that 300 mL of water does not impact on routine hematological parameters. 17 However, after breakfast, during the production of hydrochloric acid by the parietal cells in the stomach, these cells extract chloride anions, carbon dioxide, water, and sodium cations from the blood plasma, and in turn, release bicarbonate back into the plasma after its formation from carbon dioxide and water constituents. The bicarbonate content causes the venous blood leaving the stomach to be more alkaline than the arterial blood delivered to it; producing a temporary increase in pH, the phenomenon known as “alkaline tide.” 18 19 20 Thus, the alkaline tide can directly affect MCV, then the decrease observed ( Table 2 ; Fig. 1D ) can be attributed to the efflux of electrolytes and water from erythrocytes.

Postprandial leukocytosis was properly evidenced more than 85 years ago (1932), 21 revisited by Van Oostrom et al, 22 and experimentally demonstrated by Lippi et al. 4 Moreover, the ingestion of a meal increases interleukin-6, the major chemokine responsible for LYMP recruitment. 23 MONOs are activated after food intake. 24 25 Our results are in agreement with these previous studies; we observed that WBC count and NEU progressively increased 2 and 4 hours after breakfast ( Table 2 ; Fig. 1E, F ), whereas LYMP and MONO decreased 1 hour after breakfast and progressively increased 2 and 4 hours after breakfast ( Table 2 , Fig. 1G, H ). According to Klop et al, postprandial leukocyte activation is accompanied by temporary changes in leukocyte cell population data, similarly to changes observed during various infections. 26 However, physicians still request complete blood count (CBC) without advising patients regarding the required fasting time. Furthermore, laboratory medicine professionals, motivated to eliminate fasting time based on their convenience, 27 must be aware that this represents more risks than benefits for patients. 28

Postprandial lipemia is associated with changes in inflammatory and thrombotic processes that are known to be important in the development of atherosclerosis. 29 30 Platelet (PLT) function and activation after breakfast was demonstrated by MPV, without significant changes in PLT count ( Table 2; Fig. 1I, J ). In our opinion, MPV results from samples collected avoiding fasting time could improperly lead physicians to consider that as a possible risk factor of microvascular or macrovascular diseases. The routine laboratory coagulation testing including aPTT, for monitoring unfractionated heparin, 31 could be jeopardized by breakfast ( Table 3 ; Fig. 1K ). Thus, fasting time should be required before hemostasis testing.

In our study design, each volunteer was devised to be her/his own control (i.e., the results from 1, 2, and 4 hours after breakfast were compared with basal results of the same individual). Indeed, this kind of study design—a case–crossover study—is most suitable for outcomes where the induction time is short, like our evaluation of breakfast impact on laboratory test results. 32 In fact, in a case–crossover study, only cases showing discordant exposure status in the case/control window-of-time contribute to the effect and thus to the measure estimation. Because cases and controls are the same individuals, the problem of between-person confounders—that anyway exists with a control group—being constant the characteristics, do not occur. 33 Therefore, our design minimizes variability that could jeopardize the preanalytical evaluation on hematology and coagulation laboratory testing. On the other hand, large data collection is not recommended to evaluate preanalytical variability—for example, assessing fasted and nonfasted subjects using questionnaires during weeks, months, or years then correlating these information with their laboratory results—since this kind of study design appears unable to control and exclude other sources of variability as:

- Phlebotomists: each professional that performs blood collection by venipuncture has her/his own mean time of tourniquet application, and this application time is not standardized by institution, also when following international guidelines. 34 35 36
- Patient posture: the change from supine to sitting position caused clinically significant increases in the Hb, Hct, and RBC count. Furthermore, the change from supine to standing caused clinically significant increases in the Hb, Hct, RBC, WBC, NEU, LYMP, basophil, PLT, and MPV counts, and the change from sitting to standing caused clinically significant increases in Hb, Hct, RBC, WBC, NEU, and LYMP counts. 6
- Lot-to-lot variability from evacuated tubes: Lippi et al evaluated the filling of three different lots from three different sodium citrate evacuated tubes producers. Briefly, Greiner tubes were the most accurate (bias, –1.0 to 2.4%), followed by Kima (bias, –7.8 to –5.9%), and Becton Dickinson (bias, –9.6 to 3.3%) tubes. The highest between-lot difference was noted for Becton Dickinson tubes (up to 12.9%), followed by Greiner and Kima tubes (up to 3.4 and 1.8%, respectively). 37

Manufacturers of evacuated tubes worldwide commercialize three ethylenediaminetetraacetic acid (EDTA) preparations: Na _2_ EDTA, K _2_ EDTA, and K _3_ EDTA. The International Council for Standardization in Hematology recommended K _2_ EDTA (i.e., 1.5–2.2 mg/mL of blood) for hematological testing. 38 However, laboratory professionals frequently select these evacuated tubes based on costs or local availability without considering that a change of tubes could be a source of laboratory variability. 39 40 We decide to evaluate the impact of fasting time on CBC using both (K _2_ EDTA and K _3_ EDTA) in two different analytical platforms (instruments) to provide scientific evidence mirroring the “real life” in different laboratories. Moreover, the direction of interference of lipemia varies relative to parameter and analytical platform. Furthermore, the degree of bias due to the nonfasting state depends on many different mechanisms (patient metabolism, body composition, rate of food absorption, type of food, etc.). 28 Our results have shown significant differences between K _2_ EDTA and K _3_ EDTA for Hct and MCV ( Table 2 ; Fig. 1C, D ). The differences observed ( Table 2 ; Fig. 1 ) between different instruments comparing K _2_ EDTA versus K _3_ EDTA were previously described. 41 42 Obviously, different combinations between evacuated tubes and instruments could be verified by each laboratory, as required during the accreditation process. 3

It is recognized that a fasting state prior to testing is not always possible or achievable, especially in emergency settings and inpatients receiving continuous parenteral nutritional support. Therefore, clinicians should be aware of the fasting state of the patient when interpreting these diagnostic results.

When looking at the above results, these parameters might be regarded as clinically irrelevant. However, such a conclusion would be wrong with respect to the current quality specifications for bias, derived from biological variation ( Fig. 1 ). Quality managers of medical laboratories consider the quality specifications derived from biological variation 43 both very important and useful in daily practice. 44 Moreover, we are waiting anxiously for the new database regarding biological variation that are under construction on behalf of the European Federation of Laboratory Medicine. 45

In conclusion, the significant variations observed in several hematological parameters, and aPTT due to breakfast feeding demonstrate that the fasting time needs to be carefully considered prior to performing routine hematological and coagulation testing to avoid interpretive mistakes of test results, and to guarantee patient safety. Due to the abovementioned reasons, the establishment of a mathematical correction through an algorithm directed to avoid fasting time is impossible and not recommended. Therefore, COLABIOCLI WG-PRE-LATAM encourages general practitioners, nurses, technicians, and laboratory professionals to operate all together to reduce laboratory variability and to standardize the fasting requirements in their laboratory (i.e., 12 hours) using the evidence reported above.
